# Exposing an “Intangible” Cognitive Skill Among Collegiate Football Players: III. Enhanced Reaction Control to Motion

**DOI:** 10.3389/fspor.2019.00051

**Published:** 2019-10-30

**Authors:** Scott A. Wylie, Brandon A. Ally, Nelleke C. van Wouwe, Joseph S. Neimat, Wery P. M. van den Wildenberg, Theodore R. Bashore

**Affiliations:** ^1^Department of Neurosurgery, University of Louisville, Louisville, KY, United States; ^2^Department of Psychology/Amsterdam Brain and Cognition (ABC), University of Amsterdam, Amsterdam, Netherlands; ^3^School of Psychological Sciences, University of Northern Colorado, Greeley, CO, United States

**Keywords:** compatibility, reaction time, football, athletes, executive, attention, cognition

## Abstract

Football is played in a dynamic, often unpredictable, visual environment in which players are challenged to process and respond with speed and flexibility to critical incoming stimulus events. To meet this challenge, we hypothesize that football players possess, in conjunction with their extraordinary physical skills, exceptionally proficient executive cognitive control systems that optimize response execution. It is particularly important for these systems to be proficient at coordinating directional reaction and counter-reaction decisions to the very rapid lateral movements routinely made by their opponents during a game. Despite the importance of this executive skill to successful on-field performance, it has not been studied in football players. To fill this void, we compared the performances of Division I college football players (*n* = 525) and their non-athlete age counterparts (*n* = 40) in a motion-based stimulus-response compatibility task that assessed their proficiency at executing either compatible (in the same direction) or incompatible (in the opposite direction) lateralized reactions to a target's lateral motion. We added an element of decision uncertainty and complexity by giving them either sufficient or insufficient time to preload the response decision rule (i.e., compatible vs. incompatible) prior to the target setting in motion. Overall, football players were significantly faster than non-athlete controls in their choice reactions to a target's lateral motion. The reactions of all participants slowed when issuing incompatible counter-reactions to a target's lateral motion. For football players, this cost was reduced substantially compared to controls when given insufficient time to preload the decision rule, indicating that they exerted more efficient executive control over their reactions and counter-reactions when faced with decision uncertainty at the onset of stimulus motion. We consider putative sources of their advantage in reacting to a target's lateral motion and discuss how these findings advance the hypothesis that football players utilize highly-proficient executive control systems to overcome processing conflicts during motor performance.

## Introduction

American football requires reactions to stimulus events that must be executed with exceptional speed, accuracy, and precision. Slight indecision can produce significant performance costs. Converting simple, isolated stimulus events into actions is not the fundamental challenge confronting the football player, however. Rather, it is executing reactions that require extracting critical information from a rapidly-changing chaotic visual environment in which distraction, misdirection, and unpredictability are routinely used by opponents to create momentary conflict and confusion. This added layer of processing complexity directly impacts the speed at which a player must process and translate imperative stimulus events into optimal response outputs. The working hypothesis that guides our research is that the executive cognitive control systems engaged to resolve these processing conflicts are more proficient among elite football players than non-athletes. From our perspective, rather than being universally faster at performing basic stimulus-response operations, the executive cognitive control systems of football players more effectively shield stimulus-response processing from the constant barrage of conflicting stimulus inputs and response activations that compete for and interfere with the selection and execution of optimal response decisions. These enhanced cognitive control systems may undergird many of the on-field observations football experts (e.g., coaches, scouts) and fans anecdotally associate with the “intangible” or “instinctual” elements of a football player's skill set.

That stimulus-response operations in athletes who compete in reactive/interceptive sports like football are not universally faster than non-athletes or athletes from other sports on basic measures of simple or choice reaction times (RT)^1^ is well-established. They are sometimes faster than, comparable to, or slower than controls in their overall response speeds across different laboratory-based simple and choice reaction tasks (see relevant studies listed in Table 1). An important motivation for using global measures of simple or two-choice RT has been to identify and quantify an advantage among athletes in basic cognitive processing speed. The idea that derives from this assumption is that athletes are faster than non-athletes when reacting to the mere presentation of a stimulus or when making the most elementary decisions in response to stimulus events, and that individual differences at this rudimentary level of processing are universally predictive of decision-making speed during athletic performance across all levels of the decision-making process. However, while overall measures of simple or choice RT can serve as building blocks of decision speed in certain real-life situations that require a speeded response to a stimulus event, they may not serve as proxies for changes in the speed of components of a decision-making process as stimulus and response demands are increased.

**Table 1:** Reaction time studies.

**Study**	**Sport(s)**	**Competition level**	**Task**	**Relative overall speed**
Hülsdünker et al., 2016	Badminton	Nat/Inter	Contrast pattern reversal	Faster
Bianco et al., 2017a,b	Boxing, fencing, TT, VB	Pro	Go/nogo	Faster
Kida et al., 2005	Baseball	Univ/Pro	Go/nogo	Faster
Ottoboni et al., 2015^a^	Boxing	Amateur	Simon	Faster
Wang et al., 2013	Badminton	Univ/Pro	VSA/VSWM	Faster
Wang et al., 2017	Badminton	Univ/Pro	Eriksen	Faster
Yamashiro et al., 2013	Baseball	Exp/Univ	Simple Rx	Faster
Di Russo et al., 2006	Fencing	Nat/Inter	Discrim Rx	Faster
Zhang et al., 2015	Fencing	Nat/Inter	Go/nogo	Faster
Muiños and Ballesteros, 2013	Kung fu	Pro	VFA/FT	Faster
Hung et al., 2004	TT	Nat	Cued Rx	Faster
Kokubu et al., 2006	VB	Univ	Dual	Faster
Yamashiro et al., 2013	Baseball	Exp/Univ	Go/nogo	Comparable
Nakamoto and Mori, 2008	Baseball	Univ	Simple Rx	Comparable
Kida et al., 2005	Baseball	Univ/Pro	Simple Rx	Comparable
Di Russo et al., 2006	Fencing	Nat/Inter	Simple Rx	Comparable
Wylie et al., 2018	Football	Univ—DI	Eriksen	Comparable
Bashore et al., 2018	Football	Univ—DI	Simon	Comparable
Sanchez-Lopez et al., 2014	Martial Arts	Skilled	Go/nogo Cued go/nogo	Comparable
Kokubu et al., 2006	VB	Univ	Simple Rx	Comparable
Ottoboni et al., 2015^a^	Boxing	Amateur	Simon	Slower

*Reactive/interceptive athletes vs. controls*.

a *Used 2 variants of the Simon task; TT, table tennis; VB, volleyball; Nat/Inter, National/International; Pro, Professional; Univ/Pro, University/Professional; Exp/Univ, Experienced/University; Univ—DI, University/NCAA Division I; VSA/VSWM, visual spatial attention/visual spatial working memory; FT, finger tapping speed; Rx, reaction*.

A large body of research has revealed, for example, that simple or choice RT speed tells very little about how fast reactions can be inhibited because the neurocognitive systems mediating going and stopping are independent and dissociable (Aron and Poldrack, 2006; Ridderinkhof et al., 2011). An athlete who reacts quickly to simple stimulus events may or may not stop ongoing actions quickly. Similarly, introducing more complex visual demands (e.g., adding distractors) or response decision rules (e.g., variations in S-R mappings) to a choice reaction causes complex delays in processing speed that may or may not correlate with decision speed in the basic reaction (Miller and Ulrich, 2013). Thus, an athlete's overall decision speed in simple or choice reactions may offer little, if any, insight into individual differences that emerge as more complex processing demands are added to the decision-making process. Hence, it is critical to build a complete account of an athlete's processing speed capabilities by systematically increasing the variety and complexity of the demands imposed on that processing, both within and across a broad range of cognitive processes thought essential to performance in a given sport, and then determining the relative contributions of the various constituent components to the overall change in speed as demands increase.

We have reported that football players do not show a universal advantage over non-athletes in global RT in two basic variants of a two-choice reaction (Bashore et al., 2018; Wylie et al., 2018). Each task was designed, however, to also isolate a specific component of the decision-making process and determine the extent to which football players differ, if at all, from non-athletes in that component. Thus, our previous and ongoing research efforts go beyond using global measures of RT in search of a basic processing speed difference. We use highly-refined, laboratory-based RT tasks that have evolved from pioneering research in *mental chronometry* by the Dutch physiologist F.C. Donders and the founding father of experimental psychology at the University of Leipzig, Wilhelm Wundt, in the last 35 years of the nineteenth century (Boring, 1950; O'Shea and Bashore, 2012). These tasks have been developed to discover, quantify, and map specific cognitive processes onto specific stimulus and response processing demands. We selected tasks in our first two studies that are widely considered to engage unique processing demands that activate very specific executive control processes involved in shielding stimulus-response processes from the interfering effects of distraction (i.e., *interference control*; Wylie et al., 2018) and in suppressing incorrect response impulses (i.e., *impulse control*; Bashore et al., 2018). Participants in both studies were required to make choice reactions to a pre-determined target feature (direction of a target arrow, fill color of a target circle) that appeared and remained briefly at a specific location on the screen. Overall response speeds and accuracies of football players and non-athletes were comparable in each study. However, Wylie et al. (2018) found that football players, especially defensive players, excelled at *interference control*, and Bashore et al. (2018) found that football players, especially offensive players, excelled at *impulse control*. In both studies, we also found variations in control proficiency across the different player-position groups (e.g., quarterback, lineman, running back, linebacker, defensive back).

Our first two studies, like the large majority of basic laboratory studies in the extant literature, investigated differences between athletes and controls in responding to static stimuli (i.e., those presented at a fixed location). An obviously critical component of excelling in reactive/interceptive sports is great skill at timing movements of the entire body, parts of the body (as in American football, international soccer), or extensions of the body such as a tennis racket, baseball bat, or cricket bat to coincide with the arrival of a target object (e.g., opponent player, ball). Despite the centrality of responding with speed and accuracy to moving objects for athletes who compete at higher levels in reactive/interceptive sports, very little basic laboratory research has been devoted to determining whether these athletes are more proficient than non-athletes or athletes in non-reactive/-interceptive sports at processing and responding to motion-based stimulus information and none of this research has involved American football players. A foundational study of the extraordinary skill required to time the arrival of an extension of the body, a cricket bat, with the arrival of a high velocity external target object, a cricket ball, by McLeod (1987) found that (i) international-level cricketers adapted their swings to abrupt, unpredictable movements of pitched balls at speeds well within the range of times achieved by non-athletes in basic laboratory tasks, 190–240 ms, and (ii) cricketers and non-athletes estimated the landing locations of bowled balls with equivalent speed and accuracy. These findings suggested to McLeod that skilled cricket batsmen “…at the fundamental level … of visual reaction time … seem little different from people who lack this remarkable skill. Their skill, it seems, lies in how they use visual information to control motor actions once they have picked it up, not in the more elementary process of picking it up” (p. 58). This conclusion was prescient. Following McLeod's (1987) work, conclusions closely-related to his have been reached in studies that have found performance advantages among elite reactive/interceptive athletes relative to controls, either non-athlete or athletes who compete in non-reactive sports, when reacting to abrupt changes of a target's movement in either coincident timing [(French national- and international-level tennis) (Le Runigo et al., 2005, 2010); (Japanese university-level baseball) (Nakamoto and Mori, 2012); (French international-level table tennis) Ripoll and Latiri, 1997] or visual tracking [(French national- and international-level tennis) Mallek et al., 2017] tasks.

In football, a player's choice reactions when blocking or tackling are commonly tightly bound to timing his arrival at a point of intersection with a target, his opponent. His decisions are typically based on both the target's directional motion and abrupt changes in its motion. On-field reactions by football players to very rapid changes in a target's velocity and/or directional movement commonly correspond to reactions to abrupt changes in an opponent's lateral motion. For example, when a linebacker prepares to tackle an approaching running back, he may be faced with a choice to react to his left or to his right to mirror the sudden lateral motion of the running back and maintain an effective tackling position. Similarly, an offensive lineman's attempt to block an opponent often depends on choice lateral reactions that mirror the lateral movements of the opponent he must block. Thus, choice reactions players make on the field are frequently based on responding with speed and accuracy in the direction of (i.e., compatible with) very abrupt changes in lateral motion by opponents. Football players issue reactions that are not only compatible with the direction of an opponent's lateral motion, however, but are also counter-reactions in the opposite (i.e., incompatible) direction of the target opponent's lateral motion. The linebacker in the earlier example may first encounter an offensive lineman aiming to block him. In this situation, the linebacker may choose a counter-reaction in the opposite direction of a blocker's lateral motion to evade being blocked. Thus, proficient selection and execution of compatible and incompatible reactions to motion constitute an essential core of the basic choice reactions football players must routinely make on the field.

### The Current Study

Research dating to the seminal work of Paul Fitts on S-R compatibility (e.g., Fitts and Seeger, 1953; Fitts and Deininger, 1954) has shown that stimuli appearing in the left (*right*) visual half-field are responded to faster and more accurately with the spatially-compatible left (*right*) hand than with the spatially-incompatible right (*left*) hand (see edited volume on S-R compatibility by Proctor and Reeve, 1990). Similarly, speeded decisions are more efficient when movements are spatially compatible than when they are spatially incompatible with the lateral motion of a visual target (Michaels, 1988; Proctor et al., 1993; Ehrenstein, 1994). The increase in reaction time and decrease in accuracy associated with issuing spatially incompatible directional reactions, commonly referred to as the *cost of incompatibility*, have been attributed to the extra time needed to override the more natural tendency to issue spatially compatible responses to stimulus motion and to recode the stimulus motion to activate the less natural counter-reaction (e.g., Hommel, 1997). Indeed, overriding more natural or compatible stimulus-response associations and recoding them to incompatible ones is a key component of human executive control subject to considerable individual differences, which strikes us as a foundational skill underlying the ability to improvise one's reactions flexibly on the football field. To our knowledge, just one study has investigated a variant of stimulus-response compatibility effects in highly-skilled reactive/interceptive athletes (*university-level baseball*, Nakamoto and Mori, 2008) and no studies have investigated motion-based compatibility effects in highly-skilled football athletes.

Our conceptualization of the demands placed on football players to process and react with speed and accuracy to moving targets, in combination with the demonstrated advantage of elite reactive/interceptive athletes over controls in responding to abrupt changes in a target's directional movement and the findings from our previous research that football players as a group are more proficient than non-athletes at two types of executive control when responding to static stimulus events, led us to hypothesize that football players excel at processing and reacting to motion relative to their age counterparts and, given the centrality of reacting to motion on the field, this excellence would be expressed as a global advantage in response speed, irrespective of the compatibility of the response required. To test this hypothesis, we compared the performances of football players and non-athlete controls on a laboratory-based cognitive task that required them to make either compatible or incompatible responses to the abrupt onset of a target's lateral motion. Given the demand placed on cognitive control when incompatible, counter-reactions must be executed, we hypothesized further that the advantage would be more evident in players when an incompatible rather than a compatible response was required (i.e., the *cost of incompatibility* would be reduced relative to controls).

Not only must football players be proficient at executing compatible and incompatible reactions, they must also be able to make their response output decisions with extraordinary quickness as a play unfolds. Accordingly, we extended our hypothesis to assert that football players are most proficient relative to controls when the time available to make a response output decision is tightly constrained. To test this component of the hypothesis, we varied the time players and controls had to load the response decision rule before a target set into lateral motion. When the decision for issuing a compatible or an incompatible response can be loaded prior to a target's lateral motion, the preparation time it affords speeds reactions to the target's motion; conversely, if this decision must be made coincidental with the target's lateral motion, the extra time required to select the decision rule, load it, and process the target motion simultaneously slows decision speed (de Jong, 1995; Stoffels, 1996; Jennings et al., 2002). To optimize performance in the latter condition, one of response decision uncertainty, the critical target feature must be identified, the decision rule it signals must be determined, the target's directional motion must be perceived, and the correct response to that directional motion must be selected in parallel. Thus, this component of our hypothesis led to the prediction that the imposition of these concurrent processing demands on executive control would expose a distinct advantage among football players over non-athletes in controlling a counter-reaction to target motion that would produce the largest reduction in the *cost of incompatibility* among players.

To summarize, our hypothesis supported the following set of predictions. Football players would show a decided advantage over controls in (1) their overall reaction speed to the lateral motion of a target, as a result of faster response speeds when making either compatible or incompatible responses, particularly in (2) their speed at issuing motion-based counter-reactions (i.e., reduced *cost of incompatibility*), and (3) this advantage would be especially pronounced when issuing motion-based counter-reactions in situations calling for concurrent processing of a response decision rule signaled by a target property and its lateral motion. Support for these predictions would reveal a processing speed advantage among football players when reacting to lateral motion, particularly when challenged by the need to select and load a decision rule and activate a response concurrently, that would buttress the notion that high-level football players possess exceptional executive cognitive control systems and advance it by demonstrating that this advantage becomes increasingly manifest as demands on these systems increase. Finally, finding significant differences in our two previous studies between offensive and defensive football players as well as between different player-position groups in their proficiency at *interference control* and *impulse control*, two important executive cognitive skills, suggested to us the value of determining positional differences in executive control of responses to lateral motion. In accord with the findings from our previous research, we expected differences in executive control skills between the positional groups to be expressed in differences in reaction times to lateral motion. Specifying these differences across multiple measures of executive cognitive control would support our efforts to identify the unique cognitive demands and requisite cognitive skill sets associated with each position group on the field.

## Methods

### Participants

Data were collected from 525 male collegiate football players (mean age 20.6 ± 1.8) on current rosters of National Collegiate Athletic Association (NCAA) Division I (DI) football programs and 40 age-equivalent male controls (mean age 20.2 ± 2.3) recruited from the general student population at the University of Northern Colorado. No football player was in an active concussion protocol when tested or had experienced a blow to the head that kept him from physical activity within the prior 3 months. Controls were interviewed to confirm no history of head injury, participation in collegiate sports, or history of competitive video gaming (one prospective control participant was excluded on the basis of competing in state-level tournaments). They received course credit for their participation. All participants had normal or corrected-to-normal vision, as indicated by self-report. Informed written consent was obtained from individual controls and from athletic programs at each university where testing was conducted on behalf of its athletes. In written agreements with the athletic departments, football programs assumed all responsibilities for athlete consent to complete the protocol, and athletes were informed of the protocol, consented orally, and participated voluntarily, but were not required by the athletic department to sign a written consent. Per agreements with the athletic programs, we were granted permission to use, analyze, and report on athlete data provided that the university and athletes remained de-identified. The study and consenting procedures were reviewed and approved by the Institutional Review Boards at the University of Louisville (#16.1236) and University of Northern Colorado (#685373-6).

### Motion Compatibility Task and Procedures

The task, one of several completed by each subject at the time of testing, was administered on a MacMini that interfaced with a 17-inch Dell monitor, located on a table approximately 1 m from the participant, and an RB series response button box, positioned on the table immediately in front of the seated participant, that registered button presses with 2–3 ms RT resolution (Cedrus, Incorporated; https://cedrus.com/rb_series/). The response device was aligned with the midline of the participant's body and could be re-positioned so that his left and right index fingers rested comfortably on the far left and far right response buttons of a horizontal 7-button panel. Programming and administration of the task were accomplished using PsychToolbox and Matlab software (MathWorks, 2014).

The task was initiated when the digit 5 appeared at central visual fixation to begin a single-digit countdown, 4-3-2-1. After 1 disappeared from the screen, a 2,000 ms interval passed before a *purple*- or *yellow*-filled circle (0.6 cm diameter), luminance-matched and shown against a dark gray-colored background, appeared on the monitor screen at central visual fixation. Participants were instructed to issue either a left or a right index-finger button press based on the color of the circle and the direction it moved. The compatibility of the motion-response mapping varied with the color of the circle, but the color-response mapping rules were invariant across subjects. Our decision to keep the mapping constant across subjects was determined by a pilot study in which data were collected from 10 subjects, none of whom participated in the experimental study, to confirm that RTs to the two colors did not differ. If a *yellow*-filled circle appeared, participants were instructed to press the response button on the same side as the circle's directional movement [i.e., make a compatible (*Cp*) response]. If a *purple*-filled circle appeared, they were instructed to press the response button on the side opposite the circle's directional motion [i.e., make an incompatible (*Ip*) response]. The target was set in motion either 50 or 500 ms after it appeared, which according to previous research (de Jong, 1995; Stoffels, 1996; Jennings et al., 2002), gave participants, respectively, either insufficient or sufficient time to select and load the response decision rule. When set in motion, the color-filled circle moved along the horizontal meridian at a constant speed of 8 cm/s and disappeared from the screen 4.5 cm after it began moving. Thus, two experimental factors, *S-R Compatibility* and *Decision Load Time*, were varied, the first determined by the instructed S-R mapping between the circle's color and the compatibility of the response it signaled and the second determined by the elapsed time between the appearance of the circle and the initiation of its lateral movement. Each factor had two levels: *S-R Compatibility* (*Cp, Ip*) and *Decision Load Time* (*500, 50*).

The order of appearance of the *yellow*- and *purple*-filled circles signaling the compatibility of the response, the direction the circles moved, and the onset duration of their horizontal movement were determined pseudo-randomly; that is, with the constraint that the two colors, their directional movement, and their motion-onset time occurred with equiprobability across the task. Participants were encouraged to focus their visual attention on the center of the screen and to respond as quickly and as accurately as possible when the color-filled circle moved. From the onset of the circle's motion, they were given a 1,050 ms window to respond. After this response window elapsed, an intertrial interval of 400 ms began and ended when another *yellow*- or *purple*-filled circle appeared. Each participant completed 30 practice trials followed by 100 experimental trials. The end of the task was signaled by the appearance of a printed message, centered on the computer screen, that the task had been completed.

### Data Analyses

Mean RTs for correct response trials and mean accuracy rates were calculated for the four levels of the two experimental factors (*Cp500, Cp50, Ip500, Ip50*). However, because accuracy rates are not normally distributed in choice reaction tasks, we analyzed means of square root-transformed accuracy rates (McDonald, 2014). The costs of incompatibility were derived by subtracting mean RTs and accuracy rates for *Cp* trials from those for *Ip* trials for the two load-time durations. The costs of insufficient load time were derived by subtracting the mean RTs and accuracy rates for the 500 ms from those for the 50 ms load time for compatible and incompatible responses. Smaller costs on RT and accuracy rates indicate higher proficiency at producing incompatible responses and at activating responses when there is insufficient time to preload them. These measures were first analyzed using repeated measures analysis of variance (ANOVA) to determine the main and interactive effects of *S-R Compatibility* (*Cp, Ip*), *Decision Load Time* (*500, 50*), and *Group* [*football player (FB), general student control (GS)*]. We then completed a repeated measures ANOVA to determine the extent to which performance differences exist between players differentiated by *Position Group* [*Offense:* Quarterback (*QB, n* = 51), Running Back (*RB, n* = 50), Wide Receiver (*WR, n* = 67), Tight End (*TE, n* = 34), Offensive Lineman (*OL, n* = 90); *Defense:* Defensive Lineman (*DL, n* = 62), Linebacker (*LB, n* = 67), and Defensive Back (*DB, n* = 104)]. This analysis was followed by *post-hoc* pairwise comparisons to determine if there were differences in performance between the various positional subgroups among the football players as well as between each of these subgroups and general students.

We augmented the ANOVAs by computing select effect sizes (Cohen's *d*), 95% confidence intervals (0.95 CI), and Bayes factors (Wagenmakers, 2007; Rouder et al., 2009; Wetzels et al., 2011; Jarosz and Wiley, 2014). These supplemental analyses were restricted to differences we predicted between football players and controls that received statistical support from the ANOVAs. Cohen's *d* values and Bayes factors provide a quantitative framework within which to interpret the strength of experimental effects. Cohen's *d* values (either positive or negative in direction) <0.2 are considered small effects, values from 0.2 to 0.5 small to medium effects, values from 0.5 to 0.8 medium to large effects, values from 0.8 to 1.0 large to very large effects, and values >1.0 very large effects. Bayes factors (*BF*_+0_) provide an estimate of the odds favoring the alternative hypothesis (i.e., the experimental and control groups differ) over the null hypothesis (i.e., the groups do not differ). Values >1.0 are considered as evidence favoring the alternative hypothesis; the larger the departure from 1.0, the greater the confidence that an actual difference has been found. It is generally agreed that a Bayes factor 1.0–3.0 provides anecdotal evidence, 3.0–10.0 substantial evidence, 10.0–30.0 strong evidence, 30.0–100.0 very strong evidence, and >100.0 decisive evidence in favor of the alternative hypothesis. Values in the opposite direction (e.g., 1/3–1, 1/10–1/3 … 1/100–1/30, <1/100) provide increasing support for the null hypothesis. We used the JASP software (JASP Team, 2018) and default priors (*r* = 0.707; also see Rouder et al., 2009) to compute Bayes factors. In the Results section, these supplemental analyses are integrated with the associated ANOVA tests of the main hypotheses.

## Results

### Comparison of Collegiate Football Players and Controls

**Figure 1:**
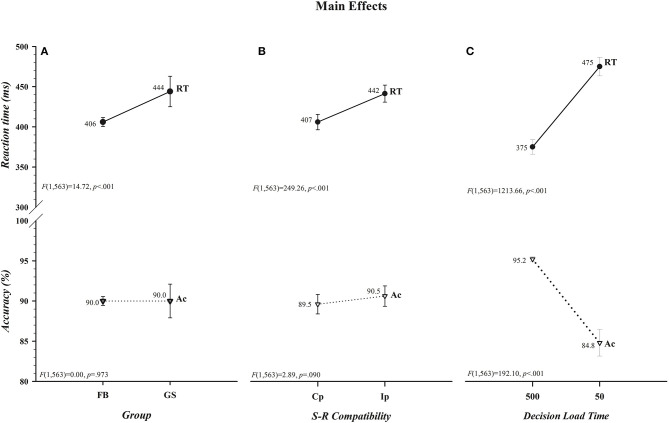
Main effects of *Group, S-R Compatibility*, and *Decision Load Time* for reaction time (RT), shown in ms, are illustrated, respectively, in the upper panels of **(A–C)**. The absence of parallel main effects for accuracy (Ac), shown in percent correct, for *Group* and *S-R Compatibility* and the presence of a main effect for *Decision Load Time* are depicted, respectively, in the lower panels of **(A–C)**. Overall, response speeds of football players (FB) were faster than, but equally accurate to, those of controls (GS), compatible (Cp) responses were faster than, but equally accurate to, incompatible (Ip) responses, and responses were slower and less accurate when *Decision Load Time* was insufficient (50) as opposed to sufficient (500). The values for each data point are shown by each point. Error bars represent the 0.95 confidence interval. Associated *F* ratios and *p*-values are shown in each panel.

The effects of variations in *Group, S-R Compatibility*, and *Decision Load Time* are illustrated in Figure 1. It can be seen in Figure 1A that, in support of the first component of our hypothesis, the overall response speed of football players to directional motion was significantly faster than that of controls (406 vs. 444 ms) and this speed advantage came at no cost to overall response accuracy (90.0% for each group) (*Group* [RT, *F*_(1, 563)_ = 14.72, *p* < 0.001] [Acc, *F*_(1, 563)_ = 0.00, *p* = 0.973]). The strength of this difference in response speed between the two groups (−38 ms, 0.95 CI [−60, −19]; *t*_(563)_ = −3.87, *p* < 0.001) was reflected in a medium to large effect size (*d* = −0.65, 0.95 CI [−0.96, −0.31]) and in a Bayes factor (BF_10_ = 171.20) indicating decisive evidence favoring the alternative hypothesis (i.e., the overall RTs of football players to a laterally-moving target are faster than those of non-athlete controls). It can also be seen in Figure 1B that there was a significant *cost of incompatibility* on response speed but not on response accuracy (*S-R Compatibility* [RT, *F*_(1, 563)_ = 249.26, *p* < 0.001] [Acc, *F*_(1, 563)_ = 2.89, *p* = 0.090]). *Ip* responses were 35 ms slower than *Cp* responses, but this slowing came at no cost or benefit to accuracy (90.5 vs. 89.5%). Unlike differences in group and compatibility effects being restricted to RT, it is clearly discernible in Figure 1C that there were decided costs of insufficient time to select and load the compatibility decision rule on both response speed and accuracy rate (*Decision Load Time* [RT, *F*_(1, 563)_ = 1213.66, *p* < 0.001] [Acc, *F*_(1, 563)_ = 192.10, *p* < 0.001]). Reactions were about 100 ms slower and 10% less accurate when participants were given insufficient (50 ms) as opposed to sufficient (500 ms) time to load the compatibility decision rule before the color-filled circle was set in motion.

**Figure 2:**
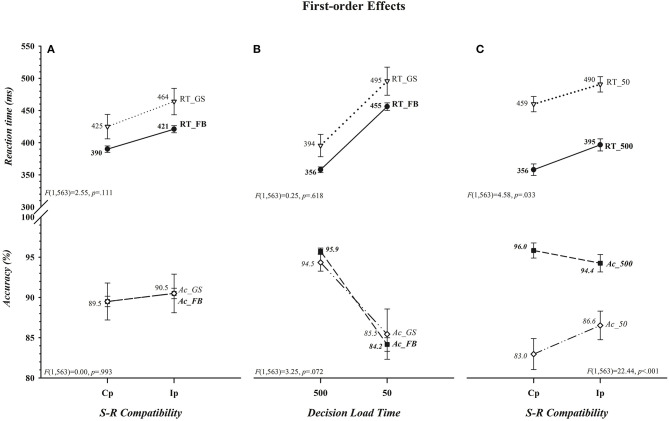
Absence of first-order differences between football players (FB) and controls (GS) in the costs of incompatibility (*Group* × *S-R Compatibility*) or of insufficient decision load time (*Group* × *Decision Load Time)* on either reaction time (RT), shown in ms, or accuracy (Ac), shown in percent correct, are depicted, respectively, in **(A,B)**. The presence of first-order benefits of incompatibility (*Decision Load Time* × *S-R Compatibility*) on both dependent measures (i.e., reductions in the cost of incompatibility on both RT and Ac) when *Decision Load Time* was insufficient (i.e., 50 ms) as opposed to sufficient (i.e., 500 ms) are illustrated in **(C)**. The values for each data point are shown by each point. Error bars represent the 0.95 confidence interval. Associated *F* ratios and *p*-values are shown in each panel.

**Figure 3:**
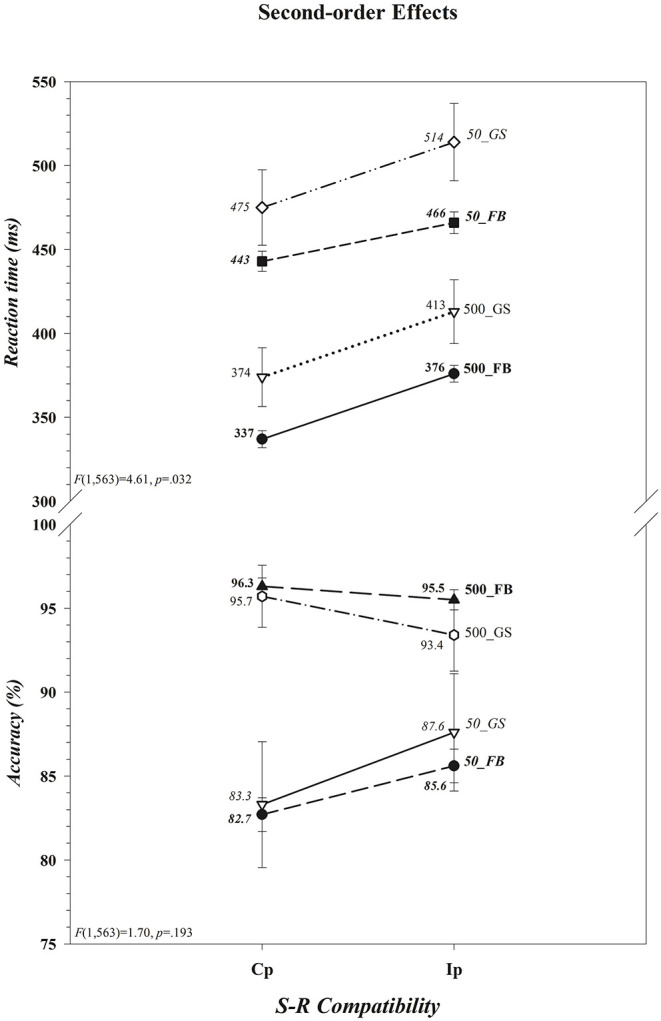
Presence of a second-order difference (*Group* × *Decision Load Time* × *S-R Compatibility*) in the *cost of incompatibility* on reaction time (RT) but not on accuracy (Ac). The *cost of incompatibility* on RT was reduced among football players (FB) but not among controls (GS) when incompatible responses were made and there was insufficient (50 ms) as opposed to sufficient (500 ms) time to load the decision rule, whereas the benefit to accuracy did not differ between the two groups. The values for each data point are shown by each point. Associated *F* ratios and *p*-values are shown in each panel. Error bars represent the 0.95 confidence interval. Cp, compatible; Ip, incompatible.

That neither the magnitude of the *cost of incompatibility* nor of the *cost of insufficient load time* differed between the two groups for either RT or accuracy is apparent in, respectively, Figures 2A,B ([*Group* × *S-R Compatibility*: RT, *F*_(1, 563)_ = 2.55, *p* = 0.111; Acc, *F*_(1, 563)_ = 0.00, *p* = 0.993] [*Group* × *Decision Load Time*: RT, *F*_(1, 563)_ = 0.25, *p* = 0.618; Acc, *F*_(1, 563)_ = 3.24, *p* = 0.072]). The absence of an overall difference between football players and controls in the size of the *cost of incompatibility* departs from the prediction of the second component of our hypothesis. There was, however, a significant impact of variations in decision load time on the *cost of incompatibility* on both RT and accuracy rate; the cost, shown in Figure 2C, reduced on RT as the accuracy rate increased (i.e., there was a *benefit of incompatibility*) when participants had insufficient as opposed to sufficient time to select and load the compatibility decision rule before the colored circle was set in motion (RT, 31 vs. 39 ms; Acc, +3.6 vs. −1.6%) (*Decision Load Time* × *S-R Compatibility* [RT, *F*_(1, 563)_ = 4.58, *p* = 0.033] [Acc, *F*_(1, 563)_ = 22.44, *p* < 0.001]). Moreover, as can be seen in Figure 3 this effect was restricted to RT and, in support of the third component of our hypothesis, was driven primarily, if not entirely, by football players (*Group* × *Decision Load Time* × *S-R Compatibility* [RT, *F*_(1, 563)_ = 4.61, *p* = 0.032] [Acc, *F*_(1, 563)_ = 1.70, *p* = 0.193]). Indeed, visual inspection of Figure 3 suggests that the *cost of incompatibility* was stable among controls across both *Decision Load Time* levels (i.e., was additive), whereas it was reduced among football players when they had *insufficient* rather than sufficient time to preload the compatibility rule (i.e., was underadditive). This visual impression was confirmed in within-group ANOVAs of the *cost of incompatibility*, calculated by subtracting the mean RT on compatible trials from the mean RT on incompatible trials for each subject (RT*Ip*–RT*Cp*). The cost was invariant in size across load times among controls (*t*_(39)_ = −0.003, *p* = 0.997; [500] 38.55 ms vs. [50] 38.58 ms; difference = <1 ms, 0.95 CI [−1.5, 1.5]), an invariance that was reflected in a negligible effect size (*d* = −0.0005, 0.95 CI [−0.31, 0.31]) and in a Bayes factor that provided substantial evidence (BF_01_ = 5.86) favoring the null hypothesis (i.e., RT costs are comparable in the two *Decision Load Time* conditions). In contrast, the cost was larger among football players when they had sufficient time (39.37 ms) as opposed to insufficient time (23.60 ms) to preload the decision rule [*t*_(524)_ = 8.075, *p* < 0.001]. This difference, 15.77 ms (0.95 CI [12, 20]), expressed a small to medium effect size (*d* = 0.35, 0.95 CI [0.26, 0.44]) and a Bayes factor that provided decisive evidence (BF_10_ = 9.3 × 10^11^) favoring the alternative hypothesis that the RT cost was smaller among football players when they had very little time as opposed to ample time to select and preload the decision rule.

Thus, there is analytic convergence in support of the hypothesis that football players are faster than their non-athlete counterparts at reacting to a target's lateral motion. Moreover, and of greater interest, there is converging analytic support for the hypothesis that football players possess enhanced cognitive control over counter-reactions in the more challenging situation that afforded them very little, if any, time to preload or prepare the decision rule about the precise type of reaction to activate. In other words, in times of response uncertainty when a decision rule must be selected and loaded in the moment or “on-the-fly,” football players are much more proficient than non-athlete controls at issuing what are likely unprepared counter-reactions that must be made in the absence of advanced preloading of the decision rule and in parallel with identifying the stimulus and activating the decision rule it signals.

### Comparison of Football Position Groups

First, we conducted a separate analysis in which football players were divided broadly into defensive and offensive position groups. While the RT and accuracy results of this analysis related to the main effects and interactions involving *Decision Load Time* and *S-R Compatibility* replicated those of the overall analysis, defensive players as a group showed two distinct performance advantages over offensive players. One, they had faster overall RTs than offensive players while maintaining similar accuracy rates when reacting to a target's lateral motion (*Group* [RT, *F*_(1, 523)_ = 8.89, *p* = 0.003] [Acc, *F*_(1, 523)_ = 2.91, *p* = 0.088]). Two, they had smaller load time costs on RT than offensive players when given insufficient rather than sufficient time to preload the decision rule (*Decision Load Time x Group* [RT, *F*_(1, 523)_ = 5.53, *p* = 0.019] [Acc, *F*_(1, 523)_ = 0.87, *p* = 0.351]). However, neither the costs of incompatibility on either RT or accuracy (*S-R Compatibility x Group*, [RT, *F*_(1, 523)_ = 1.04, *p* = 0.308] [Acc, *F*_(1, 523)_ = 2.30, *p* = 0.130]) nor the magnitude of the reduction in these costs evident when there was insufficient time to load the response decision rule differed between offensive and defensive players (*S-R Compatibility x Decision Load Time x Group*, [RT, *F*_(1, 523)_ = 2.59, *p* = 0.108] [Acc, *F*_(1, 523)_ = 2.58, *p* = 0.109]). Analyses in which controls were added to the *Group* factor revealed no departures in the magnitudes of the differences between the two position groups and controls, thus reinforcing the general differences found between football players and controls in the overall analysis.

Next, we examined the extent to which the very specific speed advantages we uncovered among football players as a group and among defensive players vis-à-vis offensive players in reacting to a target's lateral motion and in controlling unprepared counter-reactions characterized each different *Position Group* (eight factor levels: offensive position groups [*QB, RB, WR, TE, OL*]; defensive position groups [*DL, LB, DB*]). To do so, a repeated measures ANOVA was completed that replaced *Group* with *Position Group* as the between-subjects factor and excluded controls from the analysis. Here, we only describe the effects of *Position Group* as the patterns of main effects and interactions involving *Decision Load Time* and *S-R Compatibility* remained unchanged from the patterns reported above. No differences were found between the various position groups in this analysis in either overall RTs or accuracy rates (*Position Group* [RT, *F*_(7, 517)_ = 1.75, *p* = 0.096] [Acc, *F*_(7, 517)_ = 1.17, *p* = 0.321]), in patterns of effects on RTs or accuracy rates related either to variations in *S-R Compatibility* (*Position Group* × *S-R Compatibility* [RT, *F*_(7, 517)_ = 0.91, *p* = 0.498] [Acc, *F*_(7, 517)_ = 1.21, *p* = 0.294]) or *Decision Load Time* (*Position Group* × *Decision Load Time* [RT, *F*_(7, 517)_ = 1.32, *p* = 0.241] [Acc, *F*_(7, 517)_ = 0.96, *p* = 0.461]), or in the shared influences of variations in these three experimental factors on performance (*Position Group* × *Decision Load Time* × *S-R Compatibility* [RT, *F*_(7, 517)_ = 0.77, *p* = 0.616] [Acc, *F*_(7, 517)_ = 1.69, *p* = 0.109]). That is, all football position groups exhibited similar patterns of effects despite, as we describe next, showing distinguishable differences in the sizes of their performance advantages over controls.

**Figure 4:**
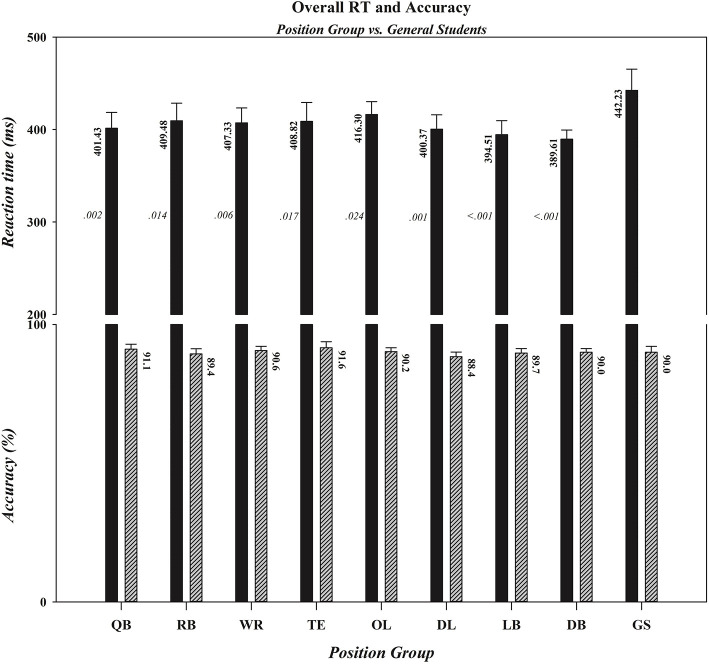
Overall RTs (in ms) and accuracy rates (in percent correct) for football players, differentiated by position, and controls (GS). Note that with the exception of tight ends, offensive linemen, and running backs the overall speed advantage was statistically significant for each position group, whereas there were no differences in overall accuracy rates between controls and any position group. Error bars represent the 0.95 confidence interval. The overall RT (shown in a black bar) and accuracy rate (shown in a diagonal-lined bar) for each group are located by the appropriate bar for that group. The *p-*value associated with each pairwise comparison between a specific position group and the control group is located by the bar depicting the overall RT for that position group. QB, quarterback; RB, running back; WR, wide receiver; TE, tight end; OL, offensive lineman; DL, defensive lineman; LB, linebacker; DB, defensive back; GS, general student control.

In the next two sets of analyses we extended the tests of the two components of our hypothesis that received statistical support in the primary ANOVAs by quantifying the degree to which the performances of individual position groups differed from the performance of controls. Specifically, we computed effect sizes and Bayes factors to determine if all or only a subset of football position groups (i) had faster overall reaction times than controls to a target's lateral motion and (ii) exhibited reduced costs of incompatibility on reaction time when given insufficient time to preload the response decision rule. As depicted in Figure 4 and summarized statistically in Table 2, the RTs of all football position groups to a target's lateral motion were numerically faster than were those of controls. However, as can be seen in Table 2, the decisiveness of the speed advantage differed across the specific position groups. Using an adjusted *p* value of .006 (accounting for the eight position-group vs. control comparisons), the *t*-tests comparing the RTs of *OL, TE*, and *RB* position groups to those of controls narrowly missed statistical significance, whereas the RT comparisons between each of the five remaining position groups (*WR, QB, DL, LB, DB*) and controls were statistically significant. Effect size and Bayes' factor analyses confirmed only anecdotal evidence supporting the response speed advantage for *OL*, but contrary to the *t*-test results, indicated substantial evidence supporting the response speed advantages for *TE* and *RB* position groups. For the remaining position groups, these additional analyses provided strong analytic convergence with the *t*-test analyses. Specifically, they indicated substantial evidence supporting the response speed advantage for *WR*s, strong evidence supporting the response speed advantages for *QB*s and *DL*, and decisive evidence supporting the response speed advantages for *LB*s and *DB*s.

**Table 2:** Differences in response speed.

**PosGrp**	**Δ**	***t*-value**	**Cohen's *d***	**Effect size**	**BF_**10**_**	**Evidentiary strength**
*OL*	−25.93 (∞, −4)	*t*_(128)_ = −2.00, *p* = 0.024	−0.38 (∞, −0.06)	Small 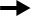 Medium	2.31	Anecdotal
*tE*	−33.41 (∞, −8)	*t*_(72)_ = −2.17, *p* = 0.017	−0.51 (∞, −0.11)	Medium	3.45	Substantial
*RB*	−33.25 (∞, −9)	*t*_(88)_ = −2.25, *p* = 0.014	−0.48 (∞, −0.12)	Medium	3.89	Substantial
*WR*	−34.90 (∞, −12)	*t*_(105)_ = −2.56, *p* = 0.006	−0.51 (∞, −0.18)	Medium 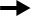 Large	7.39	Substantial
*QB*	−40.00 (∞,−18)	*t*_(89)_ = −2.94, *p* = 0.002	–0.62 (∞, −0.26)	Medium 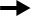 Large	17.68	Strong
*DL*	−41.86 (∞, −20)	*t*_(100)_ = −3.15, *p* = 0.001	−0.64 (∞, −0.30)	Medium 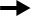 Large	30.29	Strong
*LB*	−47.72 (∞, −26)	*t*_(105)_ = −3.63, *p* < 0.001	−0.73 (∞, −0.39)	Large	119.96	Decisive
*DB*	−52.62 (∞, −35)	*t*_(142)_ = −4.92, *p* < 0.001	−0.92 (∞, −0.60)	Large	12710.05	Decisive

*Position group vs. non-athlete control group. Cohen's d, Bayes factor score*.

*PosGrp, Position Group; Δ, mean difference in RT vis-à-vis non-athlete controls; BF, Bayes factor; 0.95 CI shown in parentheses*.

Recall that the size of the *cost of incompatibility* on RT among non-athlete controls remained constant whether they were making a prepared or an unprepared counter-reaction (38.55 vs. 38.58 ms) to the target's lateral motion (i.e., *S-R Compatibility* and *Decision Load Time* were additive). In contrast, although the cost on RT among football players as a group did not differ from controls when executing a prepared counter-reaction (39.37 ms), it was reduced markedly (23.60 ms) when issuing an unprepared counter-reaction to motion (i.e., *S-R Compatibility* and *Decision Load Time* were underadditive). Notably, as summarized in Table 3, all position groups showed a reduction in RT cost in comparison to controls when making unprepared as opposed to prepared counter-reactions. Again, the magnitudes of the reduction in cost varied in their decisiveness across positions. There was anecdotal evidence supporting the cost reduction for *TE*s, which was also associated with a non-significant, marginal paired *t*-test statistic. In contrast, there was substantial evidence supporting the cost reductions for *QB*s, *DL, LB*s, and *DB*s; strong evidence supporting the cost reduction for *RB*s; and decisive evidence supporting the cost reductions for *OL* and *WR*s.

**Table 3:** Differences in cost of incompatibility-insufficient load time.

**PosGrp**	**Δ**	**Cohen's *d***	**Effect size**	**BF_**10**_**	**Evidentiary strength**
*tE*	−13.32 (∞, −1)	−0.30 (∞, −0.01)	Small 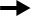 Medium	1.37	Anecdotal
*DB*	−10.80 (∞, −4)	−0.24 (∞, −0.08)	Small 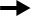 Medium	3.79	Substantial
*LB*	−10.96 (∞, −4)	−0.30 (∞, −0.10)	Small 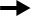 Medium	4.52	Substantial
*QB*	−18.73 (∞, −6)	−0.36 (∞, −0.12)	Small 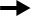 Medium	5.74	Substantial
*DL*	−16.06 (∞, −6)	−0.34 (∞, −0.12)	Small 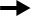 Medium	7.04	Substantial
*RB*	−20.00 (∞, −9)	−0.43 (∞, −0.18)	Small 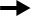 Medium	17.14	Strong
*OL*	−15.27 (∞, −8)	−0.39 (∞, −0.21)	Small 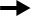 Medium	100.62	Decisive
*WR*	−24.48 (∞, −14)	−0.50 (∞, −0.28)	Small 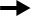 Medium	329.46	Decisive

*Position group vs. non-athlete control group. Cohen's d, Bayes factor score*.

*PosGrp, Position Group; Δ, mean reduction in cost vis-à-vis non-athlete controls; BF, Bayes factor; 0.95 CI shown in parentheses*.

In summary, the speed advantage evident among football players as a group when reacting to a target's lateral motion was preserved across the different position groups, even though the strength of this effect varied by position. Importantly, a similar pattern of performance characterized all position groups. They all showed a smaller *cost of incompatibility* when executing counter-reactions in the more demanding condition that required them to identify the target stimulus and activate the signaled response decision rule simultaneously than when they had time to select and load the decision rule in advance of the target's lateral motion. In contrast, their non-athlete counterparts showed no such advantage.

## Discussion

Choice reactions to a target's lateral motion were executed with high overall speed and accuracy. As expected, reaction times slowed significantly among all participants when they executed directionally incompatible counter-reactions rather than directionally compatible reactions to a target's lateral motion. This *cost of incompatibility* replicates longstanding findings and, more importantly, replicates a handful of studies showing that this cost is preserved using motion-based S-R compatibility tasks. As also expected, reactions were slower (by ~100 ms) and less accurate (by ~10%) when participants had insufficient (50 ms) as opposed to sufficient (500 ms) time to select and load the response decision rule before the target set in motion. This *cost of insufficient load time* replicates prior findings, confirming that selecting and loading a decision rule that guides the reaction to a stimulus is a time-consuming process. By replicating these well-established patterns of main effects, we strengthened the viability of the tests of our hypotheses about performance advantages football players possess over controls and increased confidence in the inferences we can draw from the patterns of factor effects that emerged.

### Football Players Excel in Responding to a Target's Lateral Motion

The first component of our hypothesis asserted that choice reactions to lateral motion among football players are more crucial than reacting to features of a static target and should give them an overall advantage over non-athletes in response proficiency. Consistent with this hypothesis, football players showed a decisive speed advantage over controls in reacting to a target's lateral motion (9% faster RT, comparable accuracy). Indeed, every position group produced faster RTs than controls while maintaining comparable accuracies. The magnitude of this speed advantage did vary somewhat across positions, however, with the strongest convergent analytic support revealing consistently large advantages among three defensive position groups (*DB, LB, DL*) and two offensive position groups (*QB, WR*) and slightly less consistent and smaller advantages among three offensive position groups (*RB, TE, OL*). This RT advantage contrasts with our two prior studies that found no overall advantage among football players in their choice reaction speeds to features of static targets (Bashore et al., 2018; Wylie et al., 2018). Simply adding target motion to the choice reaction produced a striking overall speed advantage for football players that was evident across all main effects and interactions and was not produced by differences in speed-accuracy tradeoffs (i.e., both groups had similar patterns of accuracy rates). How might this speed advantage be explained?

One possible explanation is that football players detect the onset of motion faster than non-athletes. That is, the speed advantage may be determined by, perhaps restricted to, differences between these groups in early motion perception. We are unaware of any studies exploring this difference between football players and either non-athlete or athlete controls. However, findings from studies of athletes who compete in other reactive/interceptive sports (badminton, baseball, soccer, tennis, volleyball) are informative. A study of dynamic visual acuity by Uchida et al. (2012), for example, revealed that Japanese university-level baseball players were more proficient than non-athlete controls at identifying the location of a gap in a moving Landolt “C” when eye movements were permitted to track its path but not when compelled to fixate a point and not move their eyes to track its motion (i.e., process foveal as opposed to peripheral motion information), suggesting that these athletes have a very early advantage in extracting details from a moving object. More broadly, the extant literature reveals that (i) experienced tennis players are more accurate, but not faster, than either athlete (triathletes) or non-athlete controls at responding to coherent motion within randomly moving dots (Overney et al., 2008); (ii) reaction times to radial motion are faster and latencies of some cognitive psychophysiological indices of neural processing speed are shorter among German national- and international-level badminton players than among non-athletes (Hülsdünker et al., 2017), although players at different levels of competition with different years of training are indistinguishable behaviorally or psychophysiologically (Hülsdünker et al., 2018); (iii) French university-level soccer players are generally faster and more accurate than non-athletes at reacting to both sport-specific biological actions (executing soccer kicks) and everyday actions (directional walking movements) across different viewing distances (Romeas and Faubert, 2015); (iv) Japanese university-level baseball, tennis, and badminton players recognize the direction of the smallest but not the largest gaps in a moving Landolt “C” faster and more accurately than non-athletes (Ishigaki and Miyao, 1993); and (v) Chinese professional volleyball players are faster than, but equally accurate to, non-athletes in responding to an identity change in one of three moving targets, irrespective of the number of distractors or changes in the color of the targets as they move (Zhang et al., 2009). From this small literature, we see that motion-based perception speed is typically faster and either as accurate or more accurate among reactive/interceptive athletes than controls. There is certainly sufficient precedent in this literature to warrant extending this line of inquiry to investigations comparing football players and controls.

It could be the case, however, that the primary mechanism underlying the advantage football players showed in reacting to lateral motion is related more fundamentally to later processing associated with their speed at determining the critical feature of the perceived target and its signal value (in our study, the color of the target and the decision rule, compatible or incompatible, it signals) before or as it goes into motion and then selecting and translating this information into the designated directional response output. In other words, the speed advantage may extend beyond an early stage of motion detection to later stages of target recognition, stimulus-response translation, and/or response selection. Perhaps the most informative work supporting such an advantage among elite reactive/interceptive athletes is found in studies of coincident timing initiated by the seminal work of McLeod (1987) whose conclusion about elite cricketers we characterized in the Introduction as prescient, “… Their skill … lies in how they use visual information to control motor actions once they have picked it up, not in the more elementary process of picking it up” (p. 58). His conclusion aligns with the findings addressing the second and third components of our hypothesis. We now turn to a discussion of those findings.

### Football Players: Enhanced Control of Counter-Reactions to Target Motion

For controls, the cost associated with issuing an incompatible response to a target's lateral motion was constant, about 39 ms, irrespective of the time given to preload the decision rule. That is, *Decision Load Time* and *S-R Compatibility* exerted additive effects on their reaction speed. According to Sternberg's (1969) seminal reasoning, the conceptual-methodological foundation for thousands of studies in mental chronometry since its proposal, two experimental factors that influence different discrete stages of processing produce main effects that are additive, whereas two factors that influence the same stage of processing produce main effects that interact. Sternberg's stage conceptualization does not have to be accepted for the interpretive value of statistical outcomes he advocates to be realized (for a penetrating review of this issue and suggested alternative processing conceptualization, see Miller, 1988). Thus, the additive relationship among controls indicates that they selected and loaded the response decision rule and subsequently translated stimulus motion into a correct response output in a sequential, stage-like fashion. Interestingly, the cognitive control needed to execute motion-based counter-reactions did not change among controls in the more challenging situation when the decision to engage this type of response control could not be made in advance of the target's movement. Football players, on the other hand, showed a different pattern of effects. When they could select the response decision rule and load it before the target moved, the cost was comparable to that of controls, about 39 ms, contrary to the prediction of the second component of our hypothesis. Thus, while football players were globally faster than controls at executing reactions to the target's lateral motion, when they had sufficient time to select and preload the response decision rule they, like controls, appear to have done so in a sequential, stage-like fashion. However, consistent with the prediction of the third component of our hypothesis, when given insufficient time to determine the response decision rule, the more challenging condition, football players excelled in their execution of incompatible counter-reactions (a 43% reduction in the *cost of incompatibility*). That is, *Decision Load Time* and *S-R Compatibility* exerted an underadditive effect on their reaction speed. According to Sternberg's reasoning, this interaction indicates that selecting and loading the response decision rule and translating stimulus motion into a correct response output occurred within a single stage. However, according to Miller (1988), when pressed for time football players activated the different components of the decision-making process in parallel.

This interaction supports the conclusion that football players possess enhanced executive control over counter-reactions to lateral motion in demanding situations when there is response decision uncertainty at the time of stimulus motion and the decision to issue a counter-reaction must be made in parallel with processing the target's lateral motion. According to a prevailing theoretical account, this interaction also indicates that football players are more proficient than controls at proactively harnessing, or s*uppressing*, their automatic stimulus-response tendencies in unpredictable situations (de Jong, 1995). Within this conceptual framework, compatibility effects result from the interplay between a “direct” or “automatic” processing route that involves rapid translation of stimuli into their strongly associated response outputs and an “indirect” or “controlled” processing route that involves slower, deliberate, rule-based translation of stimuli into appropriate goal-related response outputs (also see Ridderinkhof et al., 1995). On compatible trials, the response activated by the fast, direct route signals the response selected by the controlled processing route, which may facilitate response speed. On incompatible trials, in contrast, the controlled route must select the incompatible response as it suppresses automatic activation of the compatible response along the direct route, which slows reaction time (i.e., produces the *cost of incompatibility*) (Hommel, 1997). In prior studies of typical adults reacting to static stimuli, a reduced *cost of incompatibility* on RT is commonly reported when participants have insufficient (e.g., ≤100 ms) as opposed to sufficient (e.g., ≥500 ms) time to preload response decision rules (de Jong, 1995; Stoffels, 1996; Jennings et al., 2002). When there is contextual uncertainty about the response decision rule (e.g., compatible or incompatible S-R mappings vary randomly on a trial-by-trial basis) and/or about the time available to load the rule (e.g., sufficient or insufficient decision load times vary randomly on a trial-by-trial basis), de Jong (1995) proposed that individuals proactively suppress the direct route at task initiation to reduce performance costs on trials requiring incompatible responses. Proactive suppression of the automatic response produces opposite effects on compatible and incompatible trials. On compatible trials, it hinders the speed of selecting the response required by the decision rule. Thus, the *usual* RT advantage evident when both processing routes signal a compatible response is reduced when there is decision rule uncertainty because processing along the direct route is suppressed. In contrast, on incompatible trials, proactive suppression of the direct route lowers the activation level of the competing compatible response to benefit the speed of selecting the incompatible, counter-reaction along the indirect route. These contrasting effects yield a reduced *cost of incompatibility*; the larger the magnitude of this reduction the stronger the inferred proactive suppression of the direct route.

That non-athletes in the current study did not show a reduction in cost when provided insufficient time to preload the decision rule suggests that they did not suppress the direct route proactively when faced with contextual uncertainty about the decision rule and the amount of time to determine, select and activate the appropriate response. Football players, on the other hand, showed a significant reduction in cost when faced with such uncertainty, which suggests that they did suppress automatic stimulus-response activations proactively to better prepare for the possibility of having to issue a more difficult incompatible counter-reaction. Why is there this disparity between football players and controls in showing evidence of proactive suppression when challenged by insufficient decision load time as they reacted to lateral motion, when prior studies have shown clear evidence of this suppression in non-athlete participants when similarly challenged as they reacted to static stimuli? While we cannot answer this question conclusively, one speculation is that it may have been more difficult for controls, perhaps beyond their current capability, to suppress the strong directional response automaticity activated by the very rapid onset of the target's lateral movement. In contrast, the ability of football players to suppress these strong, automatic response activations may relate to the exceptional cognitive demands made on them to compete at an elite level on the field. At any moment, football players must react or counter-react to the uncertain, often lateral, motion and misdirection of their opponents' movements. The ability to proactively keep in check strong, automatic reactions in the direction of lateral motion is likely critical to optimizing reactions in the face of contextual and response uncertainty.

The decided advantage football players showed in reacting to abrupt, unpredictable movements of a target stimulus when given insufficient time to preload a response decision rule may share a kinship, as we argue next, with the advantages elite reactive/interceptive athletes have demonstrated in studies of coincident timing and tracking and may reflect a more global advantage in a neurocognitive skill that these athletes possess vis-à-vis non-athletes or athletes in non-receptive/-interceptive sports.

### Enhanced Executive Control Systems in Elite Reactive/Interceptive Athletes

The effects we observed, a comparable *cost of incompatibility* for football players and non-athletes when given sufficient time to load the decision rule and a reduction in cost among football players but not controls when given insufficient time to load the decision rule, are consistent with findings in studies of coincident timing and visual tracking with elite French and Japanese reactive/interceptive athletes we referenced in the Introduction (Ripoll and Latiri, 1997; Le Runigo et al., 2005, 2010; Nakamoto and Mori, 2012; Mallek et al., 2017). Revealed by these studies, without exception, are performance advantages among these athletes relative to either athlete or non-athlete controls that were evident only when the movement velocity or trajectory of an interceptive or tracking target changed abruptly and unpredictably, not when it moved at a constant velocity along a stable trajectory. As examples, the time to adjust on-going interceptive or tracking movements of a target-in-motion and the speed of that movement toward the interception or target-alignment point for a moving target in response to unpredictable deviations in its movement speed or trajectory were found to be, respectively, shorter and faster and less variable in elite French tennis players than in non-athletes (Le Runigo et al., 2005, 2010), and international-level competitors were found to be more proficient than national-level competitors at re-aligning a cursor with a moving target after perturbation of its trajectory (Mallek et al., 2017). Hence, the conclusion by Le Runigo et al. that elite tennis players are more proficient than non-athletes at making on-line adjustments linking the identity of a target stimulus to the action it signals (i.e., often referred to as visuomotor transformation or perception-action coupling) when challenged by sudden departures in target movement and the proficiency of these adjustments is associated with competitive skill level. Together, these findings indicate that elite reactive/interceptive athletes are distinguishable from other non-athlete and athlete controls by their advanced skill at translating sudden deviations in motion information into activation of motor control over the reactive adjustments demanded by these deviations.

A more finely-articulated explanation of our findings and those from the larger motion perception-action literature can be derived from the findings of Hülsdünker et al. (2017). These investigators fractionated the detection of radial motion onset into its constituent stimulus and response levels using a variety of cognitive psychophysiological measures. They found that elite German badminton players had faster overall response speeds than non-athletes, decidedly earlier onsets of EMG activation from the muscles controlling the overt response, and earlier peak latencies of two negative-going components elicited 100–300 ms after motion onset, the N200^2^ and the BA6 negativity. The former is widely-considered to reflect activation of the motion-sensitive middle temporal (MT) cortex associated with detection of motion and the latter to reflect activation of Brodmann's area 6 (BA6) in pre/supplementary motor cortex associated with transformation of a critical stimulus input into a designated response output immediately prior to movement execution (see references in Hülsdünker et al., 2017). Importantly, the earlier the peak latency of both components the earlier the EMG onset and the faster the RT, suggesting to Hülsdünker et al. (2017) that these components reflect radial motion processing in area MT that is very tightly linked to execution of the signaled overt response. Notably, component activity preceding the N200, thought to be unrelated to processing specific stimulus features, and activity originating from Brodmann's area 4, thought to reflect activation of primary motor cortex as a motor output command is sent to peripheral response effectors, were not correlated with either EMG onset or RT. Hence, the conclusion by Hülsdünker et al. (2017) that “… superior visuomotor performance in athletes is primarily related to visual perception/processing whereas motor-related processes only play a minor role” (p. 1109). In an earlier study, Hülsdünker et al. (2016) found no differences between elite badminton players and non-athletes in early ERP components evoked by perceptual processing in primary visual cortex and visual processing in extrastriate cortex when responding to a static stimulus, chromatic and achromatic contrast pattern reversal. They did, however, find earlier onsets of the BA6 negativity and of EMG activation and faster RTs among the badminton players than controls, suggesting to them that faster “visuomotor transformation” differentiated these athletes from controls.

Of immediate relevance to our findings is Hülsdünker et al. (2017) finding that EMG activation began after the peak of the N200 in non-athletes, the common observation in this population, but before the peak in athletes. This departure among athletes in the relative timing of EMG activation and N200 latency presented an explanatory conundrum to the investigators “… because a serial dependence of visual perception/processing and EMG onset would be expected” (p. 1107). The underadditive and additive effects we found between *S-R Compatibility* and *Decision Load Time* among, respectively, athletes and controls suggests an explanation for Hülsdünker et al. (2017) finding that derives from Sternberg's (1969) and Miller's (1988) reasoning. Recall our interpretation, based on this reasoning, that when given insufficient time to preload the decision rule athletes identified the critical stimulus feature, determined the decision rule it signaled, and selected the appropriate response in parallel, whereas controls completed this processing sequentially. Similarly, our interpretation of the N200 latency-EMG onset timing observed by Hülsdünker et al. (2017) is that athletes detected the onset of radial motion and activated their response to it concurrently, whereas controls detected the onset of motion and activated their response to it in seriatim.

Differences between university-level baseball players and athlete controls at modifying coincident timing actions in response to an unpredictable deceleration in target velocity reported by Nakamoto and Mori (2012) complement the findings of Hülsdünker et al. (2017). Like Le Runigo et al. (2005, 2010), and Nakamoto and Mori (2012) saw no differences in performance between players and controls when intercepting a target moving at a constant velocity. In addition, they found no differences between the two groups in the properties of two ERP components, the N200^3^ and the P300. However, advantages in motor reprogramming and interception accuracy were revealed among players when an unpredictable deceleration in the target's velocity occurred either 100, 200, or 300 ms before it reached its endpoint. The performance advantage was particularly evident at the longest interval, encouraging Nakamoto and Mori to characterize the players' performance as “… remarkably better …” (p. 28) and to restrict their analyses of ERP activity to this interval. They found that in response to target deceleration N200 latency was shorter in baseball players than in athlete controls, N200 amplitude was comparable between the two groups, and P300 latency slowed and P300 amplitude increased in players as they remained unchanged in controls. Nakamoto and Mori (2012) speculated that baseball players are more proficient than athlete controls at the type of motor reprogramming required at the two shortest intervals, automatic inhibition of the prepared response (reflected in N200 latency) and subsequent activation of the delayed response; whereas they are decidedly better at the type of controlled motor reprogramming required at the longest interval, controlled inhibition of the prepared response (reflected in P300 latency) and subsequent activation of the delayed response (see references in Nakamoto and Mori, 2012, for evidence supporting the time-related partitioning of controlled and automatic processing).

This speculation is consistent with a growing literature that argues for blurred distinctions between automatic and controlled processing in elite athletic performance (see discussion in Bashore et al., 2018). However, on the basis of findings from a meta-analysis, Albares et al. (2015) offered a set of penetrating methodological, technical, and analytical criticisms to argue that the N200 does not serve as either a reliable or valid index of response inhibition. With these criticisms in mind, our view is that the pattern of ERP activity observed by Nakamoto and Mori (2012) suggests that players recognize target movement deceleration more quickly (N200 latency) and re-calibrate the level of activation of the prepared movement to it more effectively (P300 amplitude) than do controls, while concurrently slowing translation of the stimulus input (i.e., change in target speed) into the designated response output (button press) until the time of target arrival with greater proficiency than do controls (P300 latency; see Verleger et al., 2005; Bashore et al., 2013, for discussions of the putative relationship between P300 latency and S-R translation). That is, the processing adjustment required to re-calibrate the timing of stimulus-response translation to the decelerated speed of the target may more closely approximate an automatic process in baseball players than in controls. A related process may have been engaged among the football players in our study when given insufficient time to select, load, and execute an incompatible response prior to the onset of target movement; a controlled process, suppression of the propensity to make a compatible response, may be activated with some degree of automaticity among football players but not among their non-athlete counterparts. In both instances, slowing down by baseball players and speeding up by football players, enhanced proactive control instituted at the beginning of the task may provide the meta-context within which a controlled process is executed either more efficiently or with a certain amount of automaticity by athletes, aided by more proficient stimulus-response translation. This same reasoning can be applied to the findings of Le Runigo et al. (2005, 2010).

### Position Differences: Subtle, but Consequential or Inconsequential?

Our first two studies revealed that executive skills differ in strength across the offensive and defensive position groups. All three defensive position groups, *LB, DB*, and *DL*, and one offensive position group, *WR/TE*^4^, demonstrated better skill at *interference control* (Wylie et al., 2018); whereas substantial skill was demonstrated by two offensive position groups, *OL* and *WR*, and one defensive position group, *LB*, and decisive skill was demonstrated by one offensive position group, *RB*, at *impulse control*^5^ (Bashore et al., 2018). Notably, *WR*s and *LB*s excelled in both executive skills. In the current study, defensive position groups excelled in overall reaction time to a target's lateral motion and in reducing the *cost of incompatibility* when decision load time was insufficient. Conventional statistical analyses failed to reveal specific position-group differences, but Bayes analyses uncovered very suggestive differences (see Table 3).

The latter invites speculation about and in-depth exploration of these differences. Broadly conceived, offensive and defensive players create starkly-contrasting meta-contexts (i.e., proactive control instituted at game time) in which to implement their executive skills. Offensive players establish a meta-context in which their skills are directed at deception and defensive players establish a meta-context in which their skills are directed at countering that deception. This distinction is expressed quite vividly in one-on-one confrontations between offensive and defensive players during a game. To achieve a consistent high-level of success, defensive players likely need a reaction speed advantage, particularly in their counter-reactions, as they more often play from a position of uncertainty about the offensive player's intended movements. For example, when an *LB* and an *RB* are isolated in the open field in a one-on-one confrontation the *LB*'s task is to tackle the *RB* and the *RB*'s task is to evade the *LB*. To succeed, the *LB* must exercise a high level of *impulse control* by not reacting prematurely to quick feigning, often lateral, actions by the *RB* that precede an explosive running action in, for example, the opposite direction. He must both control this impulse and react very quickly in the direction opposite the feigned action once it is reversed. The *RB* orchestrates the sequence of actions central to this confrontation and the *LB* must hold his position to counter-react to the final feigning action by the *RB* so he can time his explosive reaction to meet the *RB* at the point of attack and achieve his goal, tackling the *RB*.

The *LB* in this example has advantages in both *impulse* and *reaction control*. Our work has also revealed that *LB*s have better *interference control*. Do these executive skills comprise the essential subset an *LB* must possess to achieve success on the field, given the requisite physical skill set and meta-cognitive qualities^6^, or must he possess other executive skills as well? Asked more broadly, what is the set of cognitive skills a player must possess to succeed on the playing field at a specific position? To answer this question, we must (i) identify the executive skills that are exceptional in football players vis-à-vis non-athletes and athletes who compete in non-reactive sports; (ii) determine the patterns of executive skills that differentiate players across the various football positions; and, most importantly, (iii) isolate individual differences in these same executive skills among players in each position group that contribute most meaningfully to predicting successful on-field performance, the gold standard for every football coach.

### Limitations, Future Directions, and Concluding Comments

This is the third in a series of papers describing experiments designed to identify differences in executive cognitive skills between football players and non-athletes as well as between players who play different positions. In each study, we administered well-established, laboratory-based RT tasks to isolate very specific cognitive control processes. While this approach is limited in its ecological value it lays the groundwork for research, as we discussed in Bashore et al. (2018), designed to determine linkages between carefully isolated cognitive skills assessed in conventional laboratory tasks and cognitive skills assessed in sport-specific tasks and on-field performance (for a similar perspective, see the discussion in an influential meta-analysis by Voss et al., 2010). A limitation of our studies has been that they are restricted to comparisons of football players and non-athletes from the general student population. While this series of studies represents a first step, a direction we will likely take in our future research is to include comparisons of football players with other elite athletes in peak physical condition who compete in other reactive/interceptive and non-reactive/-interceptive sports, as well as non-athlete controls who differ in their level of physical fitness. Our goal is to advance the understanding of the unique and/or shared cognitive skills of high-level athletes across a wide-range of sports that require different types of physical skill and fitness as well as to characterize how differences in cognitive skills between athletes and non-athlete controls are tempered, if at all, by high levels of physical fitness in the latter. In our first two papers, we discussed a subset of issues and challenges in determining the extent to which the cognitive advantages we identified among football players are genetically hard-wired, developed through experience and practice, or like most cognitive functions, the result of a complex interplay between both genetic and environmental influences (see Wylie et al., 2018, p. 9, for a lengthier discussion). The extent to which cognitive skills can be trained, transferred, or developed to a high-level in football players or elite competitors in any sport and the limitations imposed upon those processes by genetic endowment is of foundational importance and remains an open issue for future research.

In the first study of the series, football players, particularly defensive players, were more proficient than non-athlete controls at *interference control* (Wylie et al., 2018) and in the second study football players, particularly offensive players, were more proficient than non-athlete controls at *impulse control* (Bashore et al., 2018) in the absence of an overall advantage among the players in response speed in either study. In both studies, choice reactions were made to static visual stimuli. In this, our third study, choice reactions were made to targets that moved rapidly and unpredictably along the horizontal meridian. The difficulty of these reactions was altered by varying both the compatibility of the response required and the amount of time available to prepare it. We reasoned that this reaction more closely approximates the choice reactions players routinely make on the field, and should be reflected in faster overall RTs among football players and more proficient execution of incompatible counter-reactions to target movement, especially when given insufficient time to prepare the reaction. Overall, football players were faster than controls and were decidedly more proficient when challenged by insufficient time to prepare a counter-reaction. Both groups executed counter-reactions with equal proficiency when challenged less by sufficient preparation time. This pattern of findings is consistent with that from coincident timing and tracking studies of elite reactive/interceptive athletes. We concluded that in situations that require extraordinarily quick reactions to movement and provide essentially no time to prepare a reaction, football players activate multiple components of the decision-making process concurrently, whereas non-athletes do not. Further, we reasoned that this proficiency suggests that football players execute these reactions with a certain degree of automaticity, particularly at the level of stimulus-response translation, whereas non-athlete controls continue to rely on controlled execution.

## Data Availability Statement

The datasets generated for this study are available on request to the corresponding author.

## Ethics Statement

Informed written consent was obtained from individual controls and from athletic programs at each university where testing was conducted on behalf of its athletes. In written agreements with the athletic departments, football programs assumed all responsibilities for athlete consent to complete the protocol, and athletes were informed of the protocol, consented orally, and participated voluntarily, but were not required by the athletic department to sign a written consent. Per agreements with the athletic programs, we were granted permission to use, analyze, and report on athlete data provided that the university and athletes remained de-identified. The study and consenting procedures were reviewed and approved by the Institutional Review Boards at the University of Louisville and University of Northern Colorado.

## Author Contributions

All authors made significant contributions to the conceptualization, design, and implementation of the experiment described in this paper. SW and TB wrote the early drafts of this paper and revised the content of the final draft in response to editorial comments by the co-authors (BA, NW, JN, WW) and reviewers. SW conducted the data analyses and received insightful guidance from the co-authors on interpreting the patterns of factor effects that emerged. NW developed the software platform that ran the experiment and the figures were constructed by TB using Sigma Plot 12.5 software.

### Conflict of Interest

The authors declare that the research was conducted in the absence of any commercial or financial relationships that could be construed as a potential conflict of interest.

## Footnotes

^1^Simple reaction tasks require that a single, repetitive, pre-determined response (e.g., button press by the index finger on the dominant hand) be made as fast as possible to the presentation of a stimulus, irrespective of stimulus properties or identity (i.e., a Donders' a-reaction). Choice reaction tasks require participants to discriminate which of two or more stimuli are presented and then select among available response options based on pre-determined mappings between stimuli and responses; thus, choice reactions involve both stimulus discrimination and response choice (Donders' b-reaction). For an historical overview of these types of reactions and the pioneering work of F. C. Donders in developing the seminal partitioning of basic speeded reactions, see O'Shea and Bashore (2012).

^2^The N200 investigated by Hülsdünker et al. is a composite component representing the average activity of a subset of scalp electrodes located over the central occipital cortex and bilateral temporoparietal cortical regions, extracted from a montage of 64 defined in the research literature using LORETA source localization and replicated by Hülsdünker et al. as originating from area MT. Two of the naming conventions for ERP components that have evolved over the years in cognitive psychophysiology use either the modal peak latency of the component (e.g., P300) or the modal numerical order of its sequential emergence in the elicited ERP componentry (e.g., P3). Hülsdünker et al. used the latter convention (e.g., N2). One of the authors (TRB) was trained in the Cognitive Psychophysiology Laboratory of the late pioneering figure in the field, Dr. Emanuel Donchin, who preferred the latency naming convention. Out of deference to Dr. Donchin, we use that convention (e.g., N200).

^3^The N200 studied by Nakamoto and Mori (2012) is not to be confused with the N200 studied by Hülsdünker et al. (2017). They are different components activated by different neurocognitive processes. Nakamoto and Mori recorded from a limited number of scalp electrode sites (Fz, Cz, Pz, F3, F4, C3, C4, P3, P4) and the N200 they studied was not determined by source localization. Rather, it was identified, as is conventionally done, by identifying the scalp site where its amplitude was largest. In agreement with the conventional ERP literature, the N200 they studied was largest in amplitude at central electrode site Cz. It should be noted that the amplitude of the P300 (and the Pd300) they studied was comparable at three frontal scalp sites, F3, Fz, and F4.

^4^Because of sample size limitations in this study we combined *WR* and *TE*. However, in the impulse control study, as in the current study, our sample sizes were large enough to separate the two positions. This partitioning will be maintained in all of our subsequent work.

^5^The language used to describe the skill levels in *impulse control* is taken from the Bayesian nomenclature. Those same descriptors are not used in describing variations in *interference control* because we did not complete Bayes analyses on those data. In replication studies of *interference control* we will augment conventional analyses with Bayes analyses.

^6^The term meta-cognitive refers to characteristics other than physical and cognitive skills that are assessed by football coaches and scouts, such as drive, work ethic, “grit” or persistence, family background, character.
